# Notes on Preparations for the Sick

**Published:** 1890-12

**Authors:** 


### Notes on Preparations for the Sick.

**Frame Food Preparations.**—FRAME FOOD COMPANY LIMITED, London.

The want of phosphates in food is said to be a crying evil, and is to be remedied by the administration of these preparations extracted from wheat bran.

The invaluable nourishment contained in the bran is lost to the multitude who eat white bread, and the consequences are seen in rickety bones, bad teeth, weak muscles and nerves, poverty of blood, &c., &c.

Advocates of whole meal tell us that we should eat the bran in our bread; but the phosphates in the bran are tenaciously held by the particles of the innutritious and indigestible woody fibre, which not only does not nourish, but which does harm to the system. Moreover, although the use of whole meal bread has been preached for years, the majority of both children and adults have an invincible dislike to it, and some are made ill by it.

The following features of interest and importance are brought out by Dr. P. F. Frankland's analysis of "Frame Food:"

In the first place, "Frame Food" is shown to be composed, to the extent of 90 per cent., of organic matter and mineral salts, there being less than 10 per cent. of moisture in the sample (taken at hazard) examined.

Secondly, both the organic and mineral matters are exceedingly rich in nutritive ingredients. Thus nearly one-third of the organic matter consists of *albuminoids*, while the remainder is mainly composed of carbo-hydrates, which are in this case present in a readily soluble form; viz., as sugar and dextrine, while less than one-third of these carbo-hydrates are present in the form of starch.

Again, the ash consists mainly of phosphate of potash, and thus is rich in two of the most highly prized mineral ingredients of food. Moreover, *practically the whole* of this ash is readily soluble in water, and is thus immediately available.

The albuminoids, also, are present in a readily soluble form, and are, for the most part, not coagulated in boiling; thus about three-fourths of the total nitrogen remains in solution even in boiling water.

"Frame Food," therefore, differs essentially from ordinary flour and other common farinaceous food stuffs—

1. In its greater richness in mineral salts, and notably in phosphoric acid and potash.
2. In its greater richness in nitrogen.
3. In the carbo-hydrates being largely in the soluble form as sugar and dextrine.

By a purely mechanical process—no chemicals are used—the woody particles of bran have been exhausted of their nutritious contents; the woody refuse is thrown away, and the pure soluble extract (containing over 10 per cent. of phosphates) is available for adding to white flour, for bread, and to every form of starchy and farinaceous food.

We have received the following preparations of "Frame Food":

**Extract Powder.**
**Infants' Diet.**
**Porridge.**
**Jelly**, for use as jam, or like malt extract.
**Biscuits.**
**Drink Syrup**, non-alcoholic.
**Pralines**, nourishing sweets.

Innumerable formulæ are given for the preparation of various articles into which the "Frame-food" powder may be introduced.

If mankind were compelled to have a diet-table limited to one kind of food, doubtless "Frame Food" would be the one to choose. We cannot but welcome the series of preparations as a useful variation in, and addition to, the dietary of the sick.

---

**Patent Refined Isinglass.**—G. P. Swinborne, London.

This has long been in use for the preparation of various dietetic articles, more or less nourishing, for the sick-room.

The scepticism with regard to the nutritive value of gelatin has not sufficed to displace it from the high position it has always held as an article of food in health and disease. The various experiments on dogs showing that life and health could not be sustained on gelatin alone, have not been considered proof that a substance so nearly allied to albumen should have little or no nutritive value. The experiments of Dr. Edward Smith, and others, have proved that gelatin is food, since it increases vital action in the same direction if not in the same degree, as albumen: these observations are quite in accord with the universal experience and belief of nonscientific people, that jelly is a valuable food. The purest kind of gelatin is isinglass from the sturgeon: its advantages over ordinary gelatin are—its lighter colour, less flavour, and greater thickening power. Its great utility lies in its culinary uses, rather than its actual nutritive value, which must, under ordinary conditions, be greatly diluted by water.

It may be taken in tea, broth, or milk. It dissolves readily in any warm liquid; all that is necessary is, to soak it for a minute or two and stir gently till it dissolves. The convalescent can use it in many ways, according to recipes enclosed in every packet.

---

**Caffyn's Liquor Carnis**—The Liquor Carnis Company Limited, London.

This comparatively recent addition to the dietary of the invalid is a highly condensed raw extract of meat containing much soluble albumen, and, therefore, is of high nutritive value.

It claims support on the following grounds:

1st. That it is peculiarly rich in nutritive material. The truth of this statement, as regards its most important albuminoids, may be verified in the simplest manner by heating to a temperature of 120° F. a small quantity in a test-tube or spoon, and noting the change consequent upon the coagulation of albuminoids previously held in solution.

2nd. That it presents its large proportion of nutriment in the natural form, so that its proteolysis and absorption are quickly carried out with a minimum of labour to the digestive apparatus.

3rd. That it causes not the slightest irritation throughout the digestive organs, and can therefore be taken even by the most weakly infant, with perfect safety.

4th. That it contains no *excess* of salts, nitrogenous waste matter, and *no added salts*.

5th. That it supplies all the nourishment required, in very small bulk—an advantage which is of no small moment in the successful management of many serious ailments.

---

**Wiesbaden Kochbrunnen Salts.**
**Natural Carlsbad Sprudel Salt.**
**Franz Josef Bitter Water.**

There is no lack of variety in waters and salines. Now that the habit of drinking hot water is so general there is no difficulty in the addition of some salt or saline to increase its efficacy. Patients are fully recognising the necessity for flushing the tissues, in order to wash away poisonous excreta; and the habit of using mineral waters at home, when it is inconvenient to visit the source, is encouraged by the increasing custom of distributing the concentrated salines in a convenient form.

The **Kochbrunnen Salt,** from Wiesbaden, dissolved in hot water is likely to be of the same utility as the water from the spring, which has a well-deserved reputation in all catarrhal conditions of the alimentary tract.

The **Crystallised Sprudel Salt** is neutral, white, and transparent, and forms hexagonal prisms of an oblong shape. Its taste is bitter and salty. It has a mildly dissolving effect.

The **Powdery Sprudel Salt,** obtained by evaporation and subsequent impregnation with the natural carbonic acid of the Sprudel, is an alkaline remedy, easily soluble, and, taken in large doses, acts as an aperient.

The Sprudel salt may be used either to assist the effects of the Carlsbad water or as a remedy by itself. In the latter case the dose is one or two teaspoonfuls, either in a dry state or dissolved in water. It dissolves rapidly and completely in warm water, and in this form a curative effect may be expected with more certainty. It is advisable to use water at a temperature of 40° to 50° R. This, however, does not exclude its being taken with cold water. If the solution becomes a little turbid, as it is likely to do with hard water, this does not affect the working of the salt at all. The best time for taking the salt is in the morning, shortly before breakfast, fasting.

